# E-selectin ligands recognised by HECA452 induce drug resistance in myeloma, which is overcome by the E-selectin antagonist, GMI-1271

**DOI:** 10.1038/leu.2017.123

**Published:** 2017-05-30

**Authors:** A Natoni, T A G Smith, N Keane, C McEllistrim, C Connolly, A Jha, M Andrulis, E Ellert, M S Raab, S V Glavey, L Kirkham-McCarthy, S K Kumar, S C Locatelli-Hoops, I Oliva, W E Fogler, J L Magnani, M E O'Dwyer

**Affiliations:** 1 Apoptosis Research Centre, Biomedical Sciences, National University of Ireland Galway, Galway, Ireland; 2 GlycoMimetics, Inc., Rockville, MD, USA; 3 Department of Hematology, Biomedical Sciences, National University of Ireland Galway, Galway, Ireland; 4 Insight Centre for Data Analytics, National University of Ireland Galway, Galway, Ireland; 5 Institute of Pathology, University of Ulm, Ulm, Germany; 6 Institute of Pathology, Heidelberg University Hospital, Heidelberg, Germany; 7 Experimental Therapeutics for Hematologic Malignancies German Cancer Research Center (DKFZ), Heidelberg, Germany; 8 Department of Medicine V, Heidelberg University Medical Center, Heidelberg, Germany; 9 Division of Hematology, Mayo Clinic, Rochester, MN, USA

## Abstract

Multiple myeloma (MM) is characterized by the clonal expansion and metastatic spread of malignant plasma cells to multiple sites in the bone marrow (BM). Recently, we implicated the sialyltransferase ST3Gal-6, an enzyme critical to the generation of E-selectin ligands, in MM BM homing and resistance to therapy. Since E-selectin is constitutively expressed in the BM microvasculature, we wished to establish the contribution of E-selectin ligands to MM biology. We report that functional E-selectin ligands are restricted to a minor subpopulation of MM cell lines which, upon expansion, demonstrate specific and robust interaction with recombinant E-selectin *in vitro*. Moreover, an increase in the mRNA levels of genes involved in the generation of E-selectin ligands was associated with inferior progression-free survival in the CoMMpass study. *In vivo*, E-selectin ligand-enriched cells induced a more aggressive disease and were completely insensitive to Bortezomib. Importantly, this resistance could be reverted by co-administration of GMI-1271, a specific glycomimetic antagonist of E-selectin. Finally, we report that E-selectin ligand-bearing cells are present in primary MM samples from BM and peripheral blood with a higher proportion seen in relapsed patients. This study provides a rationale for targeting E-selectin receptor/ligand interactions to overcome MM metastasis and chemoresistance.

## Introduction

Aberrant glycosylation is a hallmark of cancer cells, playing an important role in tumor progression.^1, 2^ Hypersialylation of glycoproteins and glycolipids has been linked to increased immune evasion, drug resistance, tumor invasiveness and vascular dissemination leading to metastases.^3^ Hypersialylation is largely the result of overexpression of sialyltransferases, which catalyze the attachment of sialic acids via different glycosidic linkages (α2-3; α2-6 or α2-8) to the underlying glycan chain.^4^ Overexpression of sialyltransferases contributes to an enhanced metastatic phenotype via the generation of selectin ligands.^5, 6^ Indeed, selectin ligand function requires the expression of Sialyl Lewis A or X (SLe^a/x^), which is synthesized by the combined action of α-fucosyltransferases, α2-3-sialyltransferases, β-galactosyltransferases and *N*-acetyl-β-glucosaminyltransferases.^7, 8, 9^ Three types of selectins have been described so far, the L-, E- and P-selectins. Selectins are type I membrane proteins composed of a N-terminus C-type lectin domain followed by an epidermal growth factor-like motif, a series of consensus repeats, a transmembrane domain and a short cytoplasmic tail.^10, 11^ By interacting with SLe^a/x^ containing glycoproteins and glycolipids, selectins are responsible for the slow tethering and rolling of leukocytes on the vascular endothelium that is the first step of leukocyte extravasation during inflammation or lymphocyte homing.^4^

In multiple myeloma (MM), P-selectin glycoprotein ligand-1 (PSGL-1), an L, P and E-selectin ligand,^12, 13, 14, 15, 16, 17, 18, 19^ has previously been shown to regulate homing to the bone marrow (BM), proliferation and resistance of malignant plasma cells to therapy mainly through its interaction with P-selectin.^20, 21^ However, in a recent study, a role of E-selectin together with P-selectin has been suggested during rolling of MM onto the BM microvasculature *in vivo*.^22^ We recently reported that overexpression of ST3 β-galactoside α2-3-sialyltransferase 6 (ST3Gal-6) is linked with BM homing, disease progression and poor outcome in MM.^23^ Because ST3Gal-6, together with ST3Gal-4, is critical to the generation of functional E-selectin ligands,^24, 25^ we postulated that ST3Gal-6 may have an important role in the synthesis of E-selectin ligands on MM cells, which could explain its negative influence on prognosis, given that E-selectin is constitutively expressed by the BM microvasculature.^26, 27^ Indeed, a proportion of MM cells from the MM1S and RPMI8226 lines demonstrated positive staining for Heca452, a monoclonal antibody that recognises SLe^x^-related structures that bind to E-selectin.^28, 29, 30, 31^ Although knocking down ST3Gal-6 completely abolished the Heca452 reactivity, it had only a modest effect on P-selectin rolling *in vitro*.^23^ This led us to extend our work to obtain a more complete understanding of the role of E-selectin and its ligands in MM biology. Our results reveal the existence of an important subpopulation of MM cells expressing functional E-selectin ligands, which appear to be enriched at relapse, implicate E-selectin binding in Bortezomib resistance *in vivo* and demonstrate the potential of a small molecule E-selectin inhibitor GMI-1271 to overcome this resistance.

## Materials and Methods

### Development of RPMI8226 and MM1S Heca452-enriched cells

Using flow sorting and immunomagnetic beads we were able to enrich MM1S and RPMI8226, respectively, for expression of Heca452 (Supplementary Methods). The Heca452-enriched cells from both RPMI8226 and MM1S were stable during routine subculturing and periodically checked for their Heca452 status. Cultured cells older than 1 month were discarded and a fresh aliquot was defrosted and used for further experiments.

### Primary myeloma samples

Patient MM samples were obtained with informed consent and ethical approval of the local Ethics Committee in accordance with the Declaration of Helsinki. Peripheral Blood (PB) mononuclear cells were separated using density sedimentation and immediately stained for flow cytometry analysis. Patient characteristics are reported in Supplementary Table 1. A retrospective single-center cohort of 132 patients with a monoclonal gammopathy was investigated by immunohistochemistry (IHC). In total, the series consisted of formalin-fixed paraffin-embedded BM (*n*=83) or soft tissue biopsies (*n*=49). BM biopsies were available from 33 patients with newly diagnosed MM (NDMM) and 50 relapsed/refractory MM (RRMM) patients who relapsed from previous lines of therapy containing at least one immunomodulatory drug and one proteasome inhibitor. The median age and disease stage at diagnosis was comparable in NDMM and RRMM patients.

### Immunohistochemistry

The Heca452 antibody (BD Bioscience, San Jose, CA, USA) was used to screen for Heca452 expression in plasma cells by IHC. Detailed methodology is provided in Supplementary Methods.

### Animal study

Tumorigenicity: MM1s parental (MM1s^par^) or the high Heca452 expressing population (MM1s^Heca452^) were injected intravenously into SCID/Beige (C.B-17/IcrHsd-Prkdc^SCID^) female mice (Harlan Laboratories, Indianapolis, IN, USA) at 5 × 10^6^, 5 × 10^5^ and 5 × 10^4^ tumor cells per mouse (*n*=8 mice per group). The mice were followed for weight loss and clinical signs of tumor progression. Lifespan was used as the primary end point to quantify tumorigenicity.

Treatment: About 5 × 10^6^ MM1s^par^ or MM1s^Heca452^ cells were injected intravenously into SCID mice. Five days post injection, mice were assigned to one of four treatment groups (*n*=8 per group): (1) Phosphate Buffered Saline q.d. for 21 days, (2) 40 mg/kg per day (q.d.) GMI-1271 for 21 days, (3) 0.75 mg/kg per day Bortezomib once a week (q.w.) for 3 weeks or (4) the combination of 40 mg/kg (q.d.) GMI-1271 for 21 days plus 0.75 mg/kg per day Bortezomib q.w. for 3 weeks. All treatments were given by intraperitoneal administration. Lifespan was used as the primary end point to quantify activity and log-rank tests determined the significance of differences between survival curves at *P*<0.05.

Mobilization: About 5 × 10^6^ MM1s^Heca452^ cells were injected intravenously into SCID mice. Thirty days post injection, mice were injected intraperitoneal with saline, 40 mg/kg GMI-1271 or 5 mg/kg AMD-3100 (CXCR4 inhibitor). Blood was collected at 1, 2, 4 and 8 h post injection and the percentage of human CD138 cells determined by flow cytometry.

### CoMMpass data set

RNA Cufflinks gene-level FPKM values of 549 patients in the IA8 cohort of the Multiple Myeloma Research Foundation (MMRF) CoMMpass trial (NCT145429) were employed to generate Kaplan–Meier plots and compared statistically using log-rank test integrated with the MMRF tool (https://research.themmrf.org and www.themmrf.org).

## Results

### E-selectin binding is restricted to a minor subpopulation of MM population identified by the Heca452 antibody

**Figure 1:**
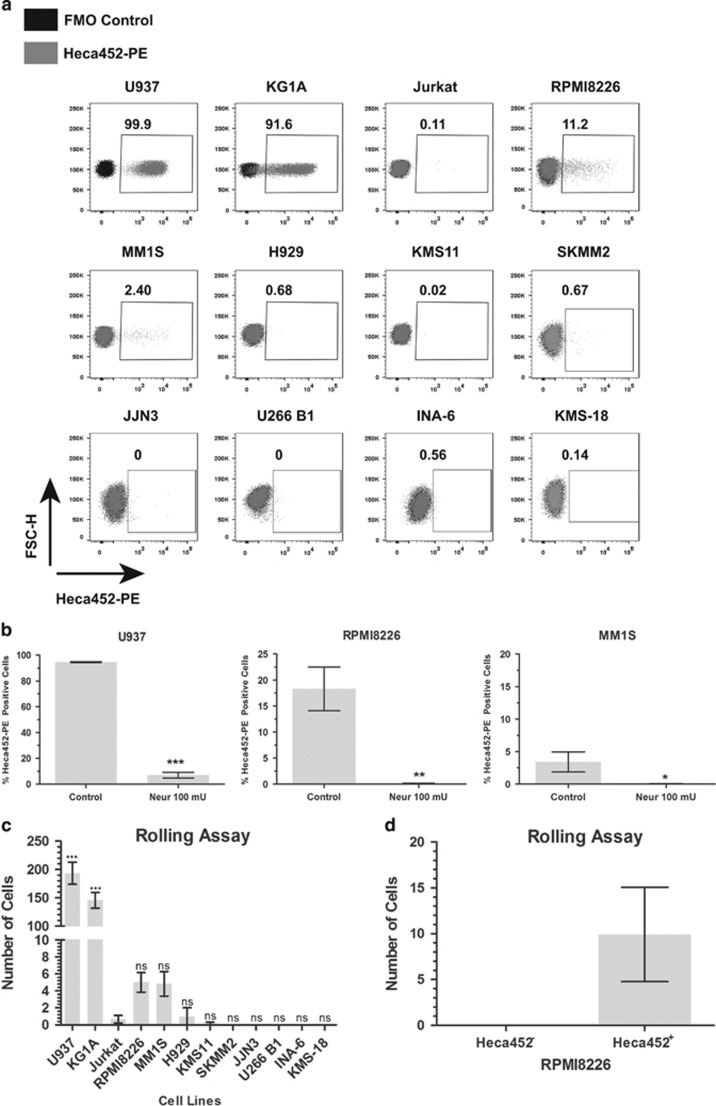
Heca452-positive myeloma cells roll on recombinant E-selectin. (**a**) The MM cell lines RPMI8226, MM1S, H929, KMS11, U266 B1, SKMM2, KMS-18, INA-6 and JJN3 together with the myeloid cell lines U937 and KG1A, and the T leukemia cell line Jurkat were stained/mock stained with the Heca452-PE antibody and analyzed by flow cytometry using the BD FACS Canto II. Data are presented as a FSC-H/Heca452-PE dot plot overlay of the fluorescence minus one (FMO, black dots) control and the Heca452-PE-stained sample (gray dots). Gates were set according to the FMO controls. Numbers indicate the Heca452-positive cells within the gates. Results are representative of at least four independent experiments. Data were acquired and analyzed using the FACS DIVA (BD Biosciences, San Jose, CA, USA) and Flow Jo 10 (FlowJo LLC., Ashland, OR, USA), respectively. (**b**) U937, RPMI8226 and MM1S were treated/mock treated with Neuraminidase (100 mU) for 45 min and then analyzed by flow cytometry as described above. Bars represent mean±s.e.m. of three independent experiments. The one tailed unpaired *t*-test was used to determine statistical significance. *, ** and *** represent *P*-values <0.05, 0.01 and 0.001, respectively. (**c**) Eighty micro liter of the indicated cell lines were loaded onto E-selectin-coated microfluidic channels and rolling assay was performed at 0.5 dyne/cm^2^ at RT using the Mirus Evo NanoPump. Rolling cells were imaged using an A-Plan × 10/0.25 objective of an A10 Vert.A1 microscope equipped with a 01 QIClick F-M-12 Mono camera. Images were acquired using the Vena Flux Assay software (Cellix Ltd., Dublin, Ireland) and analyzed using the Image-Pro Premiere (Media Cybernetics, Rockville, MD, USA). Bars represent the mean±s.e.m. of three independent experiments performed in duplicate. The one way ANOVA test following the Dunnett *post hoc* test comparing all the bars to Jurkat was used to determine statistical significance. *** represents *P*-value <0.001. (**d**) RPMI8226 cells were sorted in Heca452-positive and -negative fractions using a BD FACSAria II sorter. Rolling assay was performed on sorted cells as described above. Bars represent mean±s.e.m. of four independent experiments performed in triplicate. Statistical analysis was performed using Prism GraphPad Version 5 (GraphPad Software Inc., La Jolla, CA, USA). ANOVA, analysis of variance; FACS, fluorescence-activated cell sorter; NS, non significant.

We screened nine MM cell lines for the presence of E-selectin ligands using the Heca452 monoclonal antibody, known to bind the E-selectin carbohydrate ligand SLe^a/x^.^28^ The acute myeloid leukemia cell lines U937 and KG1A cells were used as positive controls, while the T-lymphoblast cell line Jurkat cells served as a negative control. The Heca452 antibody strongly reacted with U937 and KG1A, and did not bind Jurkat cells (Figure 1a). In MM cell lines, a minority of RPMI8226 and MM1S cells showed positivity for Heca452 (11.2% and 2.4%, respectively), while all the other MM cell lines were negative (Figure 1a); moreover, the staining pattern was quite heterogeneous with cells spanning between 10^2^ and 10^4^ log in fluorescence intensity. Importantly, Neuraminidase treatment, which removes sialic acid residues, abolished Heca452 reactivity in MM cells as well as in U937 indicating that Heca452 recognises sialic acid containing epitopes (Figure 1b).

We next evaluated the E-selectin binding activity of the cell lines under shear stress, which mimics physiologic blood flow. U937 and KG1A rolled efficiently on recombinant E-selectin, while rolling of Jurkat cells was virtually absent (Figure 1c). These results correlated well with the Heca452 profile of these cells (Figure 1a). In the MM cell lines, rolling of H929 and KMS11 cells on recombinant E-selectin was almost undetectable (Figure 1c). Rolling of RPMI8226 and MM1S cells was extremely poor suggesting that only a minor subpopulation could interact with the recombinant E-selectin. In the remaining MM cell lines, rolling cells were undetectable. Importantly, the expression levels of putative E-selectin ligands such as CD44, PSGL-1 and CD147 did not correlate with the rolling ability of MM cell lines (Supplementary Figure 1). To test whether rolling was confined to the Heca452-positive subpopulation, we sorted the RPMI8226 cell line into Heca452-negative and -positive fractions (Heca452^−^ and Heca452^+^, respectively) and repeated the rolling assay on sorted cells. Strikingly, rolling cells were only detectable in the Heca452^+^ fraction, strongly suggesting that the rolling cells consisted of the Heca452-positive cells (Figure 1d). Furthermore, when RPMI8226 cells were grown under hypoxic conditions, the percentage of the Heca452-positive cells increased (Supplementary Figure 2) suggesting an important contribution of the hypoxic microenvironment in the generation of E-selectin ligands. Taken together, these results indicate that in the MM cell lines tested, E-selectin ligands are restricted to a minority of the total population detected by the Heca452 antibody, which may be more pronounced under hypoxic conditions and suggest the specific interaction of the positive subpopulation with E-selectin.

### Stably Heca452-enriched cells display robust and specific rolling and adhesion on E-selectin

**Figure 2:**
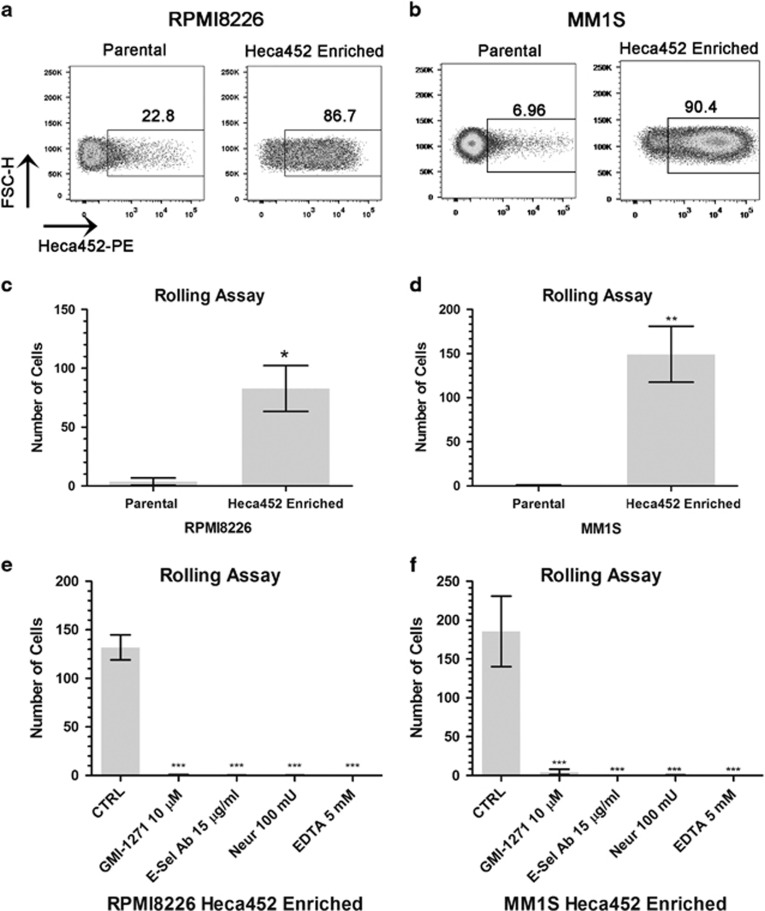
Myeloma Heca452-enriched cells display robust and specific rolling on recombinant E-selectin. FSC-H/Heca452-PE dot plot analysis of the RPMI8226 (**a**) and MM1S (**b**) parental and Heca452-enriched cells. Flow cytometry analysis was performed as described before. Data are representative of at least four different experiments. Rollin assay performed on parental and Heca452-enriched RPMI8226 (**c**) and MM1S (**d**) on recombinant E-selectin-coated channels. Rolling assay was carried out as described before. Bars represent mean±s.e.m. of three independent experiments performed in duplicate. The one tailed unpaired *t*-test was used to determine statistical significance. * and ** represent *P*-values <0.05 and 0.01, respectively. Rolling assay performed on RPMI8226 (**e**) or MM1S (**f**) Heca452-enriched cells on recombinant E-selectin-coated channels. Cells were treated/mock treated with the indicated concentration of GMI-1271 or Neuraminidase for 1 h or rolling assay was performed in the presence of 5 mM EDTA or on channels that were blocked for 1 h with 15 μg/ml of the anti-E-selectin blocking antibody. Bars represent mean±s.e.m. of four independent experiments performed in duplicate. The one-way ANOVA test following the Dunnett *post hoc* test comparing all the bars to control (CTRL) was used to determine statistical significance. *** represent *P*-values <0.001. Statistical analysis was performed using Prism GraphPad Version 5. ANOVA, analysis of variance; EDTA, ethylene diamine tetraacetic acid.

To get better insights into the biology of E-selectin ligands in MM, we established Heca452-enriched cell lines from the parental RPMI8226 and MM1S cells by labeling the cells with the Heca452 antibody and sorting the cells using either magnetic beads or fluorescence-activated cell sorter, respectively. Flow cytometry analysis of the cell lines obtained from these procedures, termed RPMI8226 and MM1S Heca452 enriched, demonstrated high enrichment of cells expressing the carbohydrate determinant recognised by the Heca452 antibody (⩾ 80% in both cell lines), which was stably maintained during subculturing (Figures 2a and b). We then performed *in vitro* rolling assays on recombinant E-selectin to functionally assess the phenotype of the Heca452-enriched cells. In accordance with their Heca452 status, a significantly higher proportion of RPMI8226 and MM1S Heca452-enriched cells showed efficient rolling on recombinant E-selectin compared to their parental counterparts (*P*=0.028 and 0.0047, respectively, Figures 2c and d). The rolling of the enriched cells on recombinant E-selectin was specific as it could be inhibited by an E-selectin blocking antibody (*P*<0.001; Figures 2e and f). Pre-treatment of the Heca452-enriched cells with ethylene diamine tetraacetic acid or Neuraminidase completely abolished rolling, demonstrating that the interaction with the recombinant E-selectin was calcium and sialic acid dependent (*P*<0.001; Figures 2e and f). Importantly, the small glycomimetic molecule GMI-1271, which inhibits the interaction between E-selectin and E-selectin ligands, blocked rolling on E-selectin in both RPMI8226- and MM1S-enriched cells (*P*<0.001; Figures 2e and f). Similar results were also obtained under static conditions (Supplementary Figure 3A).

To analyze the growth properties of the parental and the Heca452 variants, we performed proliferation experiments using cell counting and the Cell Titer Glow assay, which measures proliferation as well as metabolic activity. In both assays, the parental and Heca452-enriched cells had comparable growth rates, which were independent of E-selectin stimulation (Supplementary Figures 4A–F). To test the clonogenic potential and stem-like properties of the Heca452-enriched cells, we performed clonogenic and side population detection assays on both parental and Heca452-enriched cells.^32^ While MM1S did not form colonies, RPMI8226 cells demonstrated clonogenic potential independent of their Heca452 status (Supplementary Figure 5A). The clonogenic potential of RPMI8226 Heca452-enriched cells was not influenced by the presence of E-selectin (Supplementary Figure 5B). Moreover, Parental and Heca452-enriched RPMI8226 cells displayed comparable side populations (Supplementary Figures 5C–F), whereas neither parental nor Heca452-enriched MM1S cells exhibited a side population (Supplementary Figures 5G–H). In addition, we looked at the mRNA expression levels of putative stem cells genes such as *POU5F1*, *SOX2* and *NANOG* in the parental and Heca452-enriched MM1S cells, and found no difference in their expression (Supplementary Figure 6A). Stimulation with recombinant E-selectin had no effect, suggesting that E-selectin does not trigger a stem cell phenotype *in vitro* (Supplementary Figure 6B). Taken together, our results indicate that MM Heca452-enriched cells express functional E-selectin ligands and exhibit enhanced rolling and adhesion capabilities on E-selectin, which are amenable to therapeutic intervention. Moreover, these Heca452-enriched cells do not exhibit an enhanced clonogenic potential or stem-like properties *in vitro*.

### Heca452-enriched cells generate a more aggressive disease and are resistant to Bortezomib *in vivo,* but this can be reverted with a specific E-selectin inhibitor GMI-1271

**Figure 3:**
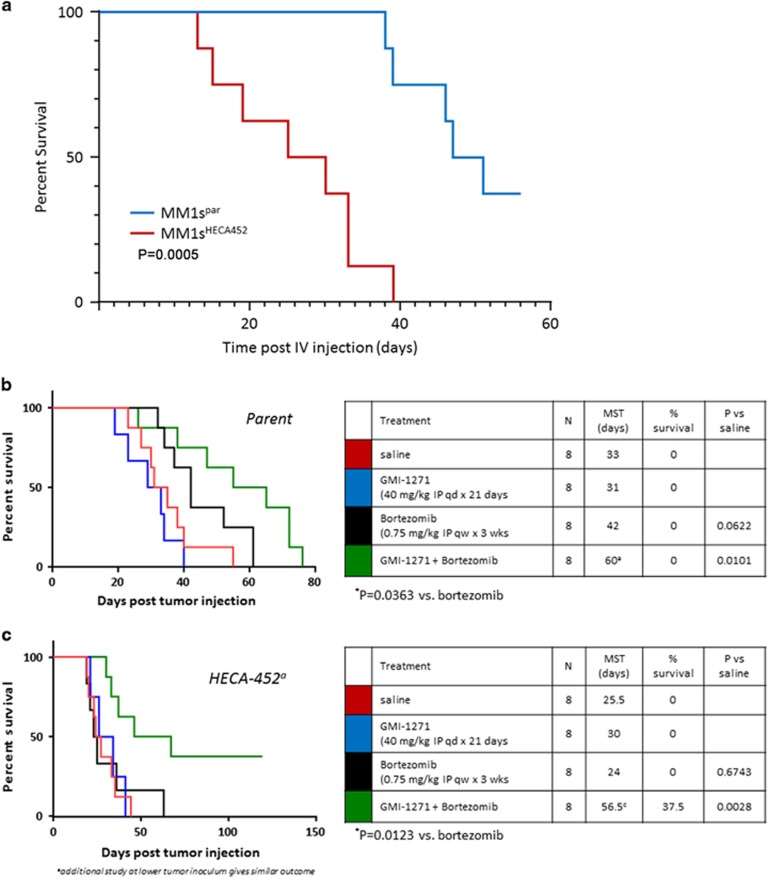
MM1S Heca452-positive cells give rise to a more aggressive disease compared to the parental cells. (**a**) Seven-week-old female scid mice were injected i.v. with 5 × 10^5^ MM1s parental (blue) or MM1s Heca452 variant (red) myeloma cells (*n*=8 mice per cell line) and the animals were observed daily for clinical signs of tumor progression (morbidity and weight loss). The results are summarized as percent survival over time. *P*-value was calculated using the Log-rank (Mantel–Cox) test. Seven-week-old female scid mice were injected i.v. with 5 × 10^6^ MM1s parental (**b**) or MM1s Heca452 variant (**c**) myeloma cells and beginning 5 days post tumor injection were treated i.p. over 21 days with daily saline alone (red line); daily 40 mg/kg GMI-1271 alone (blue line); once weekly 0.75 mg/kg Bortezomib alone (black line); or the combination of GMI-1271 and Bortezomib. Mice were observed daily for clinical signs of tumor progression (morbidity and weight loss) and the results are summarized as percent survival over time. The data were analyzed for statistical significance by Tukeys multiple comparisons test. i.v., intravenously.

To assess the significance of these results *in vivo*, parental or Heca452-enriched MM1S cells were injected intravenously into SCID beige mice. Notably, mice transplanted with Heca452-enriched cells had a significant shorter survival compared to those transplanted with parental MM1S (Figure 3a). This effect is unlikely to be due to a different proliferation rate between the Parental and Heca452-enriched MM1S cells *in vivo* as they have comparable proliferation and clonogenic capacity *in vitro* (Supplementary Figures 4 and 5). In a second cohort of mice, beginning 5 days post injection the survival impact of treatment with saline, GMI-1271, Bortezomib and a combination of both was also determined. As expected, Bortezomib treatment significantly prolonged survival of mice transplanted with parental MM1S (Figure 3b). Although GMI-1271 alone did not have any effect on survival, when combined with Bortezomib led to a significant improvement in survival of the parental MM1S engrafted mice over Bortezomib alone (*P*=0.0363; Figure 3b). In contrast to the parental MM1S line, the effect of Bortezomib in mice transplanted with MM1S Heca452-enriched cells was extremely poor with a median survival time of 24 days compared to 42 days in the MM1S parental group (Figure 3c). This chemoresistance was not due to a differential sensitivity of the MM1S parental and the Heca452-enriched cells to Bortezomib as they responded similarly to the drug when tested *in vitro* (Supplementary Figure 7). Importantly, although GMI-1271 alone did not impact survival of mice transplanted with the Heca452-enriched cells, when administered in combination with Bortezomib, GMI-1271 broke the chemoresistance and significantly restored and enhanced the anti-MM activity of Bortezomib (*P*=0.0123; Figure 3c).

### GMI-1271 mobilizes Heca452-positive human MM cells from the murine BM into the PB

**Figure 4:**
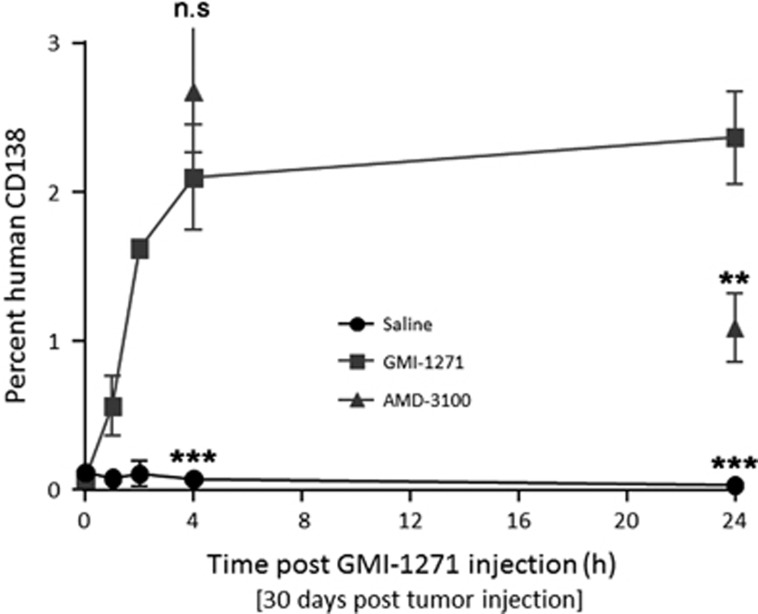
Mobilization of MM1S Heca452-enriched cells from BM to PB following GMI-1271 treatment. Seven-week-old female scid mice were injected i.v. with 5 × 10^6^ MM1s Heca452 variant myeloma cells and 30 days post tumor injection were treated i.p. with a single injection of saline, 40 mg/kg GMI-1271 or 5 mg/kg AMD-3100. Peripheral blood was collected by intracardiac puncture at 1, 2, 4 and 24 h post injection of saline or GMI-1271 (*n*=3 mice per time point) and at 4 and 24 h post injection of AMD-3100. The number of MM1s Heca452 myeloma cells was determined by flow cytometry using an anti-human CD138 antibody. Bars represent s.d. The two tailed *t*-test was used to determine statistical significance between GMI-1271 and AMD-3100, and between GMI-1271 and saline at 4 and 24 h. ** and *** represent *P*-values <0.01 and 0.001, respectively. i.v., intravenously; n.s, non significant.

To determine whether E-selectin inhibition could mobilize MM cells from the BM niche, MM1S Heca452-engrafted mice were injected with GMI-1271 and the PB was analyzed for the presence of human CD138^+^ cells. Within 60 min following a single injection of GMI-1271, the number of human CD138^+^ cells in the PB increased, and persisted increasing for at least 24 h (2.37 vs 0.03%, *P*<0.001, Figure 4). This effect was consistent with GMI-1271 disrupting the tumor microenvironment and mobilizing MM1S Heca452-enriched cells from the BM niche, with extended mobilization kinetics compared to CXCR4 inhibition.

### Heca452-positive cells are present in primary MM cells with a higher proportion in relapsed patients

**Figure 5:**
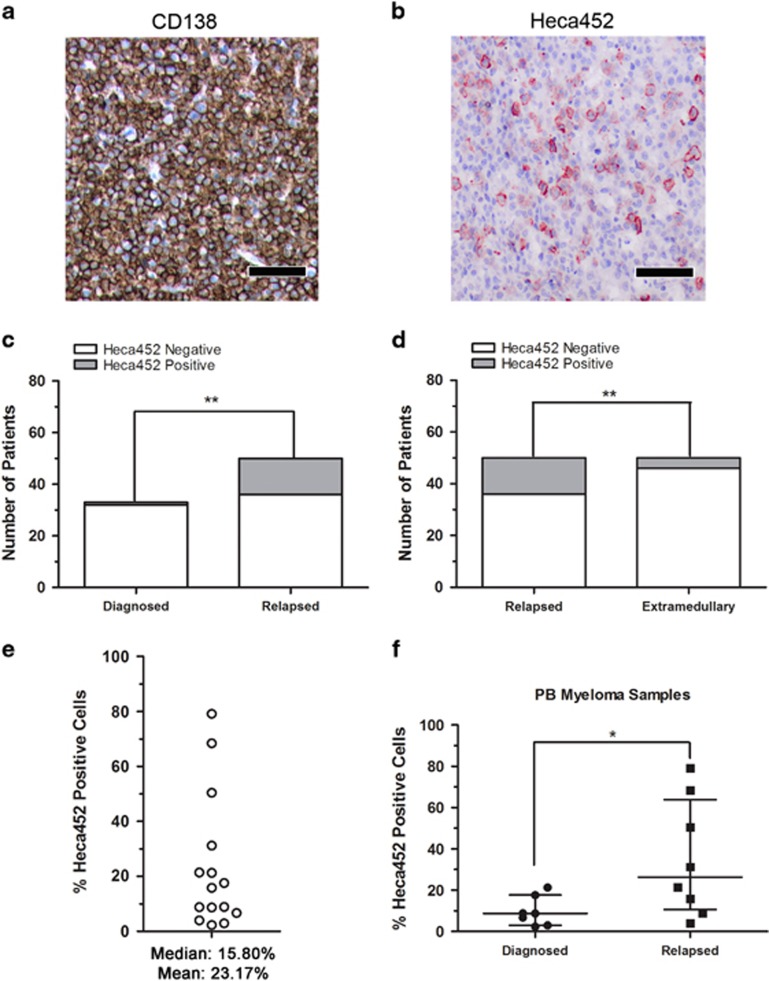
Heca452-positive cells are present within the CD38/CD138 compartment of primary myeloma cells. Representative bone marrow sections of RRMM demonstrating a high-grade infiltration by neoplastic plasma CD138-positive cells (**a**) and Heca452 expression in a subpopulation of tumor cells (**b**). Bar represents 100 μm. Heca452-positive BM biopsies in newly diagnosed vs relapsed (**c**) and relapsed vs extramedullary (**d**) patients. The *χ*^2^-test was used to determine statistical significance in the Heca452-positive cells between groups of patients. ** and * represent *P*-values <0.01 and 0.05, respectively. (**e**) Quantification of the Heca452-positive cells by FACS. Cells were first selected based on their morphology (FSC-A/SSC-A) and viability status (7AAD negativity). Cells positive for CD2, CD14 and CD235a were excluded and CD38/CD138 double-positive cells were selected to screen for the presence of the Heca452-positive cells. Each empty circle represents percentage of Heca452-positive cells within the CD38/CD138 double-positive fraction for each PB samples from myeloma patients (*N*=15). (**f**) Comparison between the percentages of the Heca452-positive cells within the CD38/CD138 double-positive fraction from the PB of diagnoses vs relapse patients. Lines represent the median with interquartile range. Statistical significance was determined using the Mann–Whitney *U*-test. * represent *P*-values <0.05. FACS, fluorescence-activated cell sorter.

Next, we evaluated the presence of MM Heca452-positive cells in primary BM samples from MM patients. Using IHC we observed Heca452 expression on a minor subpopulation (<1% of all tumor cells) of malignant plasma cells in the BM of patients with MM (Figures 5a and b). Heca452-positive plasma cells were found more frequently in BM biopsies from RRMM patients compared to NDMM patients (14/50 vs 1/33, *P*=0.009; Figure 5c). Interestingly, the Heca452-positive cells were more frequently observed in BM biopsies of RRMM patients compared to biopsies at extramedullary sites of EMM (14/50 vs 4/49, *P*=0.02; Figure 5d). The reduced ability of EMM to interact with E-selectin may reflect their increased independence from the BM microenvironment, as homing to the BM is no longer required. Next, we analyzed PB samples from MM patients for the presence of Heca452-positive malignant plasma cells by flow cytometry. To this end, the CD38/CD138 plasma cells were identified using a previously established gating strategy.^33^ Using this approach, Heca452-positive cells were consistently found in the BM and PB of patients with MM. The percentage of Heca452-positive cells in the PB was variable ranging from 2.3 to 79.10% Heca452-positive cells with a median of 15.80% (Figure 5e). Importantly, the percentage of Heca452-positive cells was higher in the PB of patients with RRMM, all of whom had relapsed following prior Bortezomib treatment (*P*=0.0289; Figure 5f). In a separate cohort, we observed that the Heca452-positive CD38/CD138 plasma cells were significantly enriched in the CD45-positive fraction, which may indicate a more immature phenotype (Supplementary Figure 8).^34^ Taken together, these data indicate that a proportion of primary MM cells express E-selectin ligands and have the potential to bind E-selectin, and these cells are more abundant in RRMM patients.

### Gene expression of genes involved in E-selectin ligand synthesis is associated with poor outcome in patients with MM

**Figure 6:**
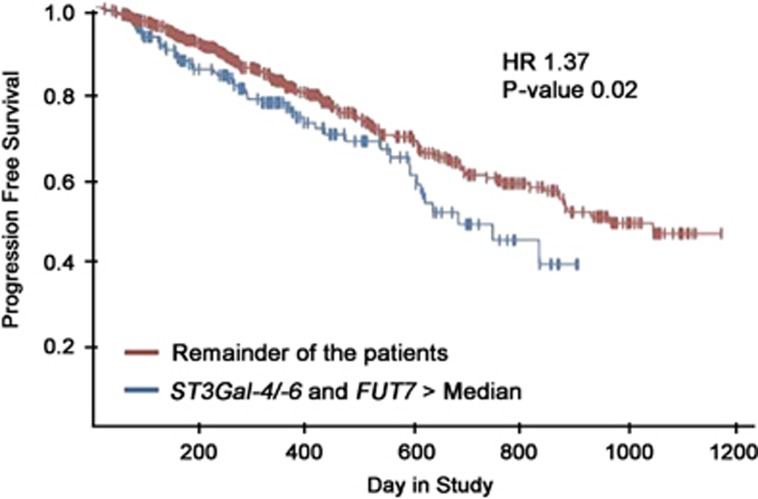
RNA expression of either *ST3Gal-4* or *ST3Gal-6* and *FUT7* greater than the median correlates with inferior survival outcomes. Kaplan–Meier estimates of PFS in MM patients with RNA expression of either *ST3Gal-4 or ST3Gal-6*, and *FUT7* greater than the median (blue) and the remainder of the patients (red) show statistically significant inferior overall PFS times (*P*-value=0.02), with a hazard ratio of 1.37. Data were generated from the CoMMpass trial. *X* axis represents time to progression in days and *Y* axis represents proportion of patients without progression. PFS, progression-free survival.

Examining RNA sequencing (seq) data from the CoMMpass study, we observed that 213 from a total of 549 patients (representing 38.8% of the cohort) exhibited RNA expression of either *ST3Gal-6 or ST3Gal-4* and *FUT7* greater than the median. These patients had significant inferior progression-free survival compared to patients with normal RNA expression of these glycosyltransferases (hazard ratio=1.37, *P*=0.02; Figure 6). Further analysis indicated this to be the case irrespective of the presence of Bortezomib in the treatment regimen (Supplementary Figure 9). This indicates poorer outcome in patients with the glycomachinery necessary to make E-selectin ligands. These findings were validated in the Shaughnessy data set (GSE2658, Supplementary Figure 10).^35^

## Discussion

Our work highlights for the first time a specific role for E-selectin and its ligands in MM. Using the Heca452 antibody, we identified a small subpopulation of MM cells capable of interacting with E-selectin. Whereas little or no rolling of unsorted MM cells was seen, Heca452-enriched MM cells rolled strongly and specifically on recombinant E-selectin. Moreover, Heca452 is the most informative marker predicting the ability of MM cells to interact with E-selectin. Indeed, all MM cell lines tested uniformly express PSGL-1 and CD147, and are variably CD44 positive, all potential glycoforms to express E-selectin ligands.^17, 30, 31, 36, 37^ Thus, it is possible that multiple known glycoprotein and/or glycolipids function as E-selectin ligands on MM cells. Despite the nature of the scaffold, our data strongly suggest that carbohydrate determinants recognised by the Heca452 antibody are required to interact with E-selectin in MM cells since E-selectin binding is sensitive to Neuraminidase treatment, which abolishes Heca452 binding. Rolling, and to a lesser extent static adhesion, on E-selectin was significantly reduced by GMI-1271, a potent and specific E- but not P-selectin inhibitor (data not shown).

The survival of mice transplanted with purified Heca452 MM1S cells was inferior to that of mice transplanted with parental MM1S cells, in keeping with our previous finding that knockdown of ST3Gal-6 improved survival.^23^ Whereas the survival of mice transplanted with parental MM1S cells was significantly improved by treatment with Bortezomib alone, the survival of mice transplanted with purified Heca452 MM1S cells was not, clearly indicating resistance to Bortezomib.

Although E-selectin is known to regulate hematopoietic stem cell dormancy in the BM niche, we were unable to show any differences in proliferation, clonogenicity or stem cell properties of Heca452-enriched vs parental cell lines. Additionally, we were unable to see any E-selectin-dependent protection of MM cells from killing by Bortezomib *in vitro* (data not shown). However, *in vivo*, E-selectin becomes an important component of Bortezomib resistance as demonstrated by the reversion of this resistance using the specific E-selectin inhibitor GMI-1271. These data also suggest that E-selectin-mediated Bortezomib resistance is purely dependent on the tumor microenvironment. Interestingly, it was recently described that E-selectin plays an important role in facilitating entry of breast cancer cells to the BM, where they are then retained by CXCR4.^38^ We believe that E-selectin may play a similar role in MM enabling the homing of MM cells to the BM in cooperation with CXCR4, inducing chemoresistance via cell adhesion-mediated drug resistance. However, MM cells are not static and are continuously trafficking through the PB from one BM site to another with the hypoxic conditions encountered by MM cells away from the vascular niche, believed to promote this dissemination.^39, 40, 41, 42^ Indeed, we observed that hypoxia markedly induced upregulation of Heca452. We speculate that inhibition of E-selectin may block the re-entry of trafficking MM cells into the BM, effectively preventing cell adhesion-mediated drug resistance. Consistent with this hypothesis, treatment with GMI-1271 mobilized MM1S cells into the PB (with extended mobilization kinetics compared to CXCR4 inhibition), but as a single agent did not improve survival in these mice. However, combination treatment with Bortezomib greatly improved survival, strongly implicating E-selectin binding in the BM as an important potential mechanism in the induction of Bortezomib resistance in MM.

The ability of GMI-1271 to overcome drug resistance was previously observed in a patient-derived acute myeloid leukemia xenograft model.^43^ A first ‘in human’ experience with GMI-1271 demonstrated favorable safety together with no mobilization of hematopoietic stem cells.^44^ On the basis of these results, a clinical trial to determine the safety, pharmacokinetics and efficacy of GMI-1271 in combination with chemotherapy in acute myeloid leukemia is currently ongoing (ClinicalTrials.gov NCT02306291) with promising results.^45^

To address the clinical significance of our findings, we examined plasma cells from patients with MM. Using IHC, a higher percentage of Heca452-positive cells was observed in BM biopsies of RRMM patients compared to NDMM patients. Significantly, the former had all relapsed following previous lines of therapy containing at least one immunomodulatory drug and one proteasome inhibitor. Similarly, in acute myeloid leukemia the expression of E-selectin ligands is higher in relapsed compared to newly diagnosed patients.^43^ Using flow cytometry, we were able to detect E-selectin ligands on MM cells from the PB and BM of both NDMM and RRMM patients. Interestingly, we observed a statistically significant increase in HECA452 positivity in the CD45-positive fraction (*P*<0.0001). CD45 is predominantly expressed by less mature plasma cells and its expression has been reported to be associated with worse outcome in MM.^34^ Moreover, the prevalence of Heca452-positive cells in the CD45-positive fraction may be favored under the hypoxic environment of the BM, which, at least in mice, seems to be advantageous for the CD45-positive MM cells.^46^ Finally, samples from RRMM patients showed a significantly higher percentage of Heca452-positive cells in the PB compared to those from NDMM patients. These data suggest a dynamic modulation of selectin ligands expression as the disease develops to a more aggressive phenotype, contributing to resistance. However, once MM cells become independent of the BM microenvironment, they may lose E-selectin ligands, as evidenced by the lower Heca452 positivity at sites of EMM. This may also explain the variable expression of Heca452 we observed in MM cell lines, as MM cell lines are derived from extramedullary disease. In contrast to our findings with IHC, by flow cytometry we did not observe a higher percentage of Heca452-positive cells in the BM of RRMM compared to NDMM (data not shown). This discrepancy might be due the different methodologies employed to identify Heca452-positive cells. The IHC may be less sensitive than flow cytometry, but has the advantage of analyzing the cells in their microenvironmental context. Thus it is possible that the Heca452 epitopes are downregulated once the cells leave the BM and are exposed to normoxic conditions (BM aspirate) as previously seen for CXCR4.^39^

We previously showed that high levels of *ST3Gal-6* gene expression were an independent risk factor for poor survival.^47^ We now report that within the CoMMpass study, 38.8% of patients have high RNA levels of either *ST3Gal-6 or ST3Gal-4* along with high levels of *FUT7*, all of which are critical genes involved in E-selectin ligand synthesis.^24, 48, 49, 50^ Thus, it is likely that these patients would have increased E-selectin ligand expression with a higher percentage of Heca452-positive MM cells. These patients have significantly inferior progression-free survival, irrespective of whether they received Bortezomib containing therapy or not, suggesting that E-selectin binding may be associated with microenvironmental drug resistance in general. Conceivably, the outcome of patients with this gene signature could be improved by targeting E-selectin and its ligands with strategies such as GMI-1271.

Overall, our data provide compelling evidence that E-selectin and its ligands play an important role in the disease progression and drug resistance in MM. There is a strong rationale for clinical strategies targeting E-selectin and its ligands to improve patient outcome in MM. This concept, evaluating GMI-1271 as an adjunct to Bortezomib-based therapy, is currently being tested in the clinic (ClinicalTrials.gov NCT02811822).
