# Supplementary material for: E-selectin ligands recognised by HECA452 induce drug resistance in myeloma, which is overcome by the E-selectin antagonist, GMI-1271
Source: Leukemia. 2017 May 30;31(12):2642–51. doi: 10.1038/leu.2017.123 (PMC5729350; doi:10.1038/leu.2017.123)
Supplement: Supplementary Information [file leu2017123x1.docx]

**Supplementary Material and Methods**

**Cell Lines**

The MM cell lines MM1S, RPMI8226, U266 B1 and H929 were from American Type Culture Collection (ATCC; Manassas, US). KMS11, SKMM2, KMS-18, INA-6 and JJN3 were kindly provided by Prof Leif Bergsagel. U937 and Jurkat cell lines were from Sigma-Aldrich (St. Louis, US) and ATCC respectively. KG1A cells were kindly provided by Prof Afshin Samali. All cell lines, including the derived variants, were grown in RPMI 1640 media supplemented with 10% heat inactivated Fetal Bovine Serum (HI FBS, Sigma-Aldrich), 50 U/ml penicillin (Sigma-Aldrich), and 50 µg/ml streptomycin (Sigma-Aldrich). INA-6 were grown in the presence of Interleukin 6 (20 ng/ml, PeProtech; Rocky Hill, US). Cell lines were maintained in a state of logarithmic growth at 37°C in a humidified incubator with 5% CO_2_ and passage in adherent 100 mm tissue culture dishes. Cells were initially authenticated by short tandem repeats (STRs) profiling and then short interspersed nuclear elements (SINE) markers profile registered for later monitoring (specific genetic markers set characterized and validated at Gentective Teoranta; Galway, Ireland). Interspecies cross-contamination including mycoplasma tests were also controlled by species specific PCRs (Gentective Teoranta). All reagents were from Sigma-Aldrich unless stated otherwise.

**Development of RPMI8226 and MM1S Heca452 enriched cells**

The MM1S Heca452 enriched cells were obtained using a MoFlo flow cytometer (DAKOCytomation; Glostrup, Denmark). MM1S cells were labelled with FITC-conjugated Heca452 (BD Biosciences; San Jose, US) for 60 min at 4°C and the Heca452 positive cells were sorted into RPMI 1640 supplemented with 20% FBS and culture at 37°C in 5% CO_2_. When the culture reached 90% confluency, the sort was repeated and the cells expanded. The newly derived cell population (MM1S^Heca452^) was enriched to 85% Heca452 positive cells.

Since RPMI8226 cells were highly sensitive to the sorting conditions, stable Heca452 positive cells were obtained using beads. Briefly, RPMI8226 were labelled with Heca452-PE conjugated antibody for 10 min at 4°C in the dark in continuous rotation and sorted using the anti-PE Multisort Microbeads (Miltenyi Biotec GmbH; Bergisch Gladbach, Germany) according to the manufacturer’s instructions. To eliminate the presence of contaminating Heca452 negative cells, sorted cells were diluted in a 96 well plate and screened for their Heca452 status. One RPMI8226 Heca452 enriched variant was selected for further experiments.

**Immunohistochemistry**

Four- to six-micrometer sections were cut from FFPE specimens and mounted on Superfrost Ultra Plus glass slides (Gerhard Menzel GmbH, Braunschweig, Germany). Tissue sections were subjected to heat-induced epitope retrieval and incubated with a primary antibody for 30 min. The signal was detected by streptavidin/3-amino-9-ethylcarbazole (AEC)-based detection Kit (LSAB II Dako, Hamburg, Germany). Cell nuclei were stained with haematoxylin (Merck, Darmstadt, Germany). The CD138 immunostaining was conducted on a Ventana BenchMark Ultra (Ventana Medical Systems, Tucson, AZ, US) automated immunostainer using a ready-to-use primary anti-CD138 antibody (clone B-A38) and standard reagents all provided by Ventana. The immunostained slides were evaluated by two pathologists. Because of the very low prevalence of Heca452 positive plasma cells, at least 10 high power fields (HPF, 400x magnification) with myeloma infiltration were screened for Heca452 expression. In majority of investigated cases the Heca452 was expressed in less than 1 plasma cell per HPF. Therefore cases with at least one Heca452 positive myeloma cell detected in the screened HPF’s were classified as positive. Microscopic figures were taken with an Olympus BX-51 light microscope equipped with a DP50-CCD camera and processed with Cell-A Software (all from Olympus, Center Valley, US).

**Rolling Assay**

Rolling assay was performed in 8 channel microfluidic biochips (Cellix Limited; Dublin, Ireland) using a Mirus Evo NanoPump (Cellix Limited). The biochip’s channels were coated with 15 µg/ml of E-selectin (PeProtech) in Tris·HCl pH 7.4 supplemented with 1 mM CaCl_2_ (coating buffer) and incubated overnight at 4°C. Each channel was blocked with 1% BSA or where indicated with 15 µg/ml of anti-E-selectin blocking antibody (Clone BBIG-E1, R&D System; Minneapolis, US) and incubated at 37°C 1 h before the assay. Cells were washed and resuspended in rolling assay buffer (RPMI 1640 media without phenol red supplemented with 1% HI FBS, 5 mM Hepes and 1 mM CaCl_2_) at 2x10^6 cells/ ml. Eighty µl of cell suspension were loaded onto the microchannels and rolling assay was run at 0.5 dyne/cm^2^ at Room Temperature (RT). Where indicated, cells were pre-treated with 100 mU Neuraminidase (Roche; Basel, Switzerland) or 10 µM GMI-1271 (GlycoMimetics Inc.; Rockville, US) for 1 h at 37°C or rolling assay was performed in the presence of 5 mM EDTA. Cells were monitored in 5 different positions along the channel using an A-Plan 10X/0.25 objective (Carl Zeiss Microscopy GmbH; Jena, Germany) of an AX10Vert.A1 Microscope (Carl Zeiss Microscopy GmbH). Thirty frames per position were collected at 0.5 sec from each other using a 01 QIClick F-M-12 Mono 12-bit camera (QImaging; Surrey, Canada). Images were acquired using the Vena Flux assay software (Cellix Limited) and the analysis was performed using the Image-Pro Premiere software (Media Cybernetics; Rockville, US). A rolling cell was defined as a cell travelling a distance corresponding to more than its diameter. The number of cells per position were added to obtain the total number of cells per channel which was then averaged between the numbers of channel.

**Adhesion Assay**

Adhesion assay under static conditions was performed in non-tissue culture treated 96 well plate. Wells were coated with 5 µg/ml of anti-human IgG Fcγ fragment specific (Jackson Immunoresearch; West Grove, US) in a total volume of 100 µl of coating buffer for 1 h at 37°C. After incubation, wells were washed twice with PBS and then coated with 5 µg/ml of recombinant human IgG_1_/E-selectin chimera (R&D System, Minneapolis, US), 5 µg/ml of recombinant human IgG_1_ Fcγ fragment (R&D System) or 1% BSA in 100 ul of coating buffer overnight at 4°C. One hour before the adhesion assay, wells were washed twice in PBS, blocked with 1% BSA or 5 µg/ml of the anti-E-selectin blocking antibody and incubated for 1 h at 37°C. Some wells were coated with 0.1% of Poly-D-Lysine for 5 min at RT, washed and then blocked for 1 h at 37°C with 1% BSA as positive controls. Cells were washed twice in PBS, resuspended at 2.5x10^6 cells/ml in RPMI 1640 media supplemented with 5 mM Hepes and 5 µM Calcein-AM (Life Technologies; Carlsbad, US) and incubated for 15 min at 37°C in a waterbath. After incubation, cells were washed twice in PBS and resuspended in assay buffer (RPMI 1640 media without phenol red supplemented with 1% HI FBS and 1 mM CaCl_2_) at 1x10^6 cells/ml. One hundred µl of cell suspension were dispensed onto the wells and the plate was incubated for 2 h at 37°C. Where stated, cells were pre-treated with 100 and 200 mU Neuraminidase, 10 and 20 µM GMI1271 or the adhesion assay was performed in the presence of 5 mM EDTA. After incubation, wells were gently washed two times with PBS supplemented with 1 mM CaCl_2_ and adherent cells were lysed in the wells using 100 µl of 1% Sodium Dodecyl Sulfate (SDS). After a 10 min incubation at RT, fluorescence was measured using a Victor^3^ 1420 multilabel counter microplate reader (Perkin Elmer; Waltham, US) with an excitation of 485 nm and a 535 nm emission filter.

**Neuraminidase Treatment**

Cells were washed and resuspended at 5x10^6/ml in RPMI 1640 supplemented with 1% HI FBS. Cells were treated with the appropriate concentration of Neuraminidase for 45 min at 37°C. After incubation, cells were washed two times and processed for further analysis.

**Flow Cytometry Analysis and FACS sorting**

Cells were washed and blocked with 5% of relevant normal serum (Jackson Immunoresearch) for 10 min on ice. After the blocking step, cells were washed and resuspended in 100 µl of staining buffer (PBS plus 1% FBS plus 5 mM Hepes) at 5x10^5 cells/sample containing the relevant antibodies and incubated for 30 min on ice. After incubation, cells were washed twice and resuspended in 500 µl of staining buffer supplemented with 0.5 µg/ml of 7-amino-actinomycin D (7AAD, Life Technologies), incubated for 5 min and analyzed immediately by a BD FACS CANTO II flow cytometer (BD Biosciences). All the gates were set according to the fluorescence minus one (FMO) control. For cell sorting, cells were first blocked and then stained with the Heca452-PE antibody as described above. Cells were washed twice and resuspended in sorting buffer (RPMI 1640 media without phenol red supplemented with 5 mM Hepes and 2% FBS) at 20x10^6 cells/ml. Cells were sorted using a BD FACSAria II sorter at 20 psi with a 100 nm nozzle. Cells were kept at 4°C in constant agitation (100 rpm). Cells were collected in RPMI 1640 media supplemented with 20% HI FBS and 5 mM Hepes. After the sorting procedure, the number of cells, viability and purity of the fractions were briefly checked and cells were processed for further analysis. The following anti-human antibodies were used in this study: PE-CLA (clone Heca452), APC- and BV421-CD38 (clone HIT2), Alexa Fluor 647-CD162 (clone KPL-1), APC-CD44 (clone G44), FITC-CD2 (clone RPA-2.10), BV421- and Alexa Fluor 647-CD138 (clone MI15), FITC-CD14 (clone M5E2) from BD Biosciences. APC-CD147 (clone 8D12) from eBiosciences (San Diego, USA). FITC-CD235a (clone HI264) from ImmunoTools GmbH (Friesoythe, Germany).

**Hypoxia Experiments**

RPMI8226 cells were grown for 5 days either under normoxic or hypoxic conditions (1% O_2_, 5% CO_2_, 37°C). After incubation, cells were collected and stained with the Heca452 antibody as described above.

**Cell Viability Assay**

MM cells were seeded onto 96 well plate at 5x10^4 and treated with different concentration of Bortezomib. After 48 and 72 h of incubation, 10 µl of the Cell Titre Glow Assay (Promega) were added per each wells. Following an incubation of 10 min at RT, luminescence was measured using a Victor^3^ 1420 multilabel counter microplate reader.

**Proliferation and Clonogenic assays**

To estimate the proliferation rate of the parental and the Heca452 enriched cells in both RPMI8226 and MM1S, cells were plated in either 12 well plates or 96 well plates at 1.5x10^5 and 3x10^6 cells/ml respectively and grown for 96 h. Cells were counted every 24 h using an Haemocytometer (for 12 well plate) or proliferation was estimated using the Cell Titer Glow assay (96 well plate). Proliferation of RPMI8226 and MM1S Heca452 enriched cells was analysed in the presence of 5 µg/ml of recombinant human IgG_1_/E-selectin chimera or recombinant human IgG_1_ Fcγ fragment in a 96 well plate format. Proliferation was estimated using the Cell Titer Glow Assay every 24 h up to 96 h. To test the clonogenic potential of the parental vs the Heca452 enriched RPMI8226 and MM1S cells lines, 0.4 ml of a 10^4/ml cell suspension were mixed with 4 ml of MethoCult H4434 (StemCell Technologies; Vancouver, Canada) and 1.1 ml of the mixture was plated in 2 ml suspension dishes using 5 ml syringes and 16 gauge needles. Plates were grown at 37°C in a humidified incubator with 5% CO_2_ for 14 days and then counted using a light microscopy. In some experiments, the RPMI8226 Heca452 enriched cells were stimulated with 5 µg/ml of recombinant human IgG_1_/E-selectin chimera or recombinant human IgG_1_ Fcγ fragment for 48 h before plating the cells for the clonogenic assay.

**Side Population**

Cells were collected, washed and resuspended in 2 ml of RPMI 1640 supplemented with 2% HI FBS and 10 mM Hepes (SIGMA) at 1x10^6 cells/ml. Cells were stained with 3 µM Hoechst 33342 (Life Technologies) in the presence/absence of 10 µM Reserpine (SIGMA) and incubated in a waterbath set at 37°C for 90 min with 30 s agitation every 30 min. After incubation, the samples were spun down at 300xg for 10 min and resuspended in HANK Salt Balance Solution (SIGMA) supplemented with 2% HI FBS, 10 mM Hepes and 0.5 µg/ml of 7AAD and analysed by Flow Cytometry using the violet laser and the 450-510-50A and 450-450-50A detectors.

**Total RNA extraction and quantitative Real Time (qRT)-PCR**

Total RNA was purified from MM cells using NucleoSpin RNA kit (Macherey-Nagel; Duren, Germany) according to the manufacturer’s instructions. One µg of total RNA was reverse transcribed using the High Capacity cDNA Reverse Transcription kit (Applied Biosystem; Carlsbad, US). The cDNA was then diluted 1/20 in RNase/DNase free water (Promega) and 5 µl were used for the qRT-PCR reaction. The qRT-PCR was carried out using the FastStart Universal Probe Mastermix (ROCHE) in a StepOne Plus Real Time PCR System (Applied Biosystem). Parameters were set as follow: activation of FastStart Taq DNA polymerase 95°C for 10 min; amplification and real time analysis 95°C for 15 sec and 60°C for 30 sec (40 cycles in total). Taqman assays used were NANOG (Hs04260336_g1), POU5F1 (Hs00999634_gh), SOX2 (Hs01053049_g1), 18S (Hs03003631_g1) and TBP (Hs00427620_m1). The 18s and TBP were used as endogenous controls to normalize the data.

Patient MM samples were obtained with informed consent and ethical approval of Clinical Research Ethics Committee University Hospital Galway in accordance with the Declaration of Helsinki

Animal Studies were performed at the Noble Life Sciences Inc. 22 Firstfield Rd, Gaithersburg, MD 20878. Human Care of Animals: Treatment of animal was in accordance with the study protocol and also in accordance with Noble SOPs which adhere to the regulations outlined in the USDA Animal Welfare Act (9 CFR Parts 1,2 and 3) and the conditions specified in the Guide of the Care and Use of Laboratory Animals (ILAR publication, NRC, 2011, The National Academies Press). The Noble Institutional Animal Care and Committee (IACUC) approved the study protocol prior to finalization to insure compliance with acceptable standard welfare and human care

**Supplementary Figure 1. Expression of putative E-selectin ligands on MM cells.** The indicated cells lines were analyzed by Flow Cytometry for the expression of CD44, CD162 and CD147. Bars represent mean ± SEM of three independent experiments.

**Supplementary Figure 2. Hypoxic conditions increase the Heca452 positive subpopulation in RPMI8226 cells.**  Cells were grown either under normoxic (21% O_2_) or hypoxic (1% O_2_) conditions for 5 days and then analysed by Flow Cytometry for the expression of the Heca452 marker. Bars represent mean ± SEM of three independent experiments. The one tailed unpaired t test was used to determine statistical significance. ** represents P value less than 0.01. Statistical analysis was performed using Prism GraphPad Version 5.

**Supplementary Figure 3. Myeloma Heca452 enriched cells display robust and specific adhesion on recombinant E-selectin.** Adhesion assay performed under static conditions on Calcein-AM labelled RPMI8226 (A) and MM1S (B) parental and Heca452 enriched cell on recombinant E-selectin/IgG chimera. Wells containing 5 µg/ml of IgG Fcγ and 1% BSA were used as negative controls. Wells coated with 0.1% Poly-D-Lysine were used as positive controls. Calcein-AM labelled cells were treated/mock treated with the indicated concentration of GMI-1271 or Neuraminidase or adhesion assay was performed in presence of 5 mM EDTA or on wells that were blocked with 5 µg/ml of the anti-E-selectin blocking antibody. After 2 h, non adherent cells were washed away and attached cells were lysed using 1% SDS for 10 min. Fluorescence was measured using the Victor^3^ 1420 multilabel counter microplate reader. Bars represent mean ± SEM of three independent experiments performed in triplicate. The one way ANOVA test following the Dunnett post hoc test comparing all the bars to Heca452 enriched E-Selectin IgG 5 µg/ml bar was used to determine statistical significance. *** and * represent P values less than 0.001 and 0.05 respectively. ns = non significant. Statistical analysis was performed using Prism GraphPad Version 5.

**Supplementary Figure 4. Parental and Heca452 enriched RPMI8226 and MM1S cells exhibit comparable proliferation independently on E-Selectin stimulation.** Parental (red line) and Heca452 enriched (black line) RPMI8226 (A) and MM1S (B) cells were counted every 24 h up to 96 h. Proliferation of parental (red line) and Heca452 enriched (black line) RPMI8226 (C) and MM1S (D) cells was assessed every 24 h up to 96 h using the Cell Titer Glow assay. The Heca452 enriched RPMI8226 (E) and MM1S (F) cells were grown in the presence of 5 µg/ml of recombinant human IgG_1_/E-selectin chimera (red line) or recombinant human IgG_1_ Fcγ fragment (black line). Proliferation was assessed every 24 h up to 96 h using the Cell Titer Glow assay. DT indicates the doubling time estimated in the exponential grow phase of RPMI8226 (72 h) and MM1S (96 h). Curves were generated using Prism GraphPad Version 5.

**Supplementary Figure 5. Parental and Heca452 enriched cells exhibit similar clonogenic potential independently on E-Selectin stimulation and comparable side population.** (A) Parental and Heca452 enriched RPMI8226 cells were plated in MethoCult H4434 on 2ml suspension dishes at 1x10^3 cell/dish in triplicate. Dishes were incubated for 14 days and colonies were counted using a light microscopy. Similar experiments were performed using the MM1S parental and Heca452 enriched cells, however no colonies were observed. (B) RPMI8226 Heca452 enriched cells were stimulated with 5 µg/ml of recombinant human IgG_1_/E-selectin chimera or recombinant human IgG_1_ Fcγ fragment for 48 h, then plated in MethoCult H4434 and analysed as described above. Bars represent mean ± SEM of three independent experiments. The two tailed unpaired t test was used to determine statistical significance. ns = non significant. Statistical analysis was performed using Prism GraphPad Version 5. Parental (C; D) and Heca452 enriched (E; F) RPMI8226 cells were treated with 10 µM Reserpine (D; F) or equal volume of DMSO (C; F) and then stained with Hoechst 33342 for 90 min at 37°C in the dark. Side population was identified by setting the gate on the population with low Hoechst 33342 staining that disappeared following Reserpine treatment. Parental (G) and Heca452 enriched MM1S (H) were analysed for the presence of the side population as described above. Representative Dot Plots from three independent experiments are presented.

**Supplementary Figure 6. The mRNA expression of putative stem cell genes do not differ between Parental and Heca452 enriched MM1S cells and are not induce following stimulation with E-selectin.** (A) Expression profile of the indicated genes for parental and Heca452 enriched MM1S cells obtained by qRT-PCR. (B) Heca452 enriched MM1S cells were stimulated with 5 µg/ml of recombinant human IgG_1_/E-selectin chimera or recombinant human IgG_1_ Fcγ fragment for 48 h. Expression profile of the indicated genes was evaluated by qRT-PCR. Data were analyzed using the StepOne Software v2.3 (Applied Biosystem). Bars represent mean ± upper/lower limit of three independent experiments performed in triplicate. Unpaired two tailed t test was used to determine statistical significance in each group of genes. ns = non significant. Statistical analysis was performed using Prism GraphPad

**Supplementary Figure 7. MM1S parental and enriched cells display overlapping sensitivity to Bortezomib *in vitro*.** MM1S parental and Heca452 enriched cells were seeded at 5x10^4 cells/ well onto a 96 well plate and treated with different concentrations of Bortezomib for 48 (A) and 72 h (B). Cell viability was measured by the Victor^3^ 1420 multilabel counter microplate reader using the Cell Titre Glow Assay. Symbols represent the mean ± SEM of three independent experiments performed in triplicate. Data were normalized to parental CTRL. Prism GraphPad Version 5 was used to generate dose responses

**Supplementary Figure 8. Primary Heca452 positive Myeloma cells are enriched in the CD45 positive fraction.** BM CD138 CD38 double positive plasma cells from Myeloma patient were analysed for CD45 and Heca452 expression by flow cytometry. Lines represent the median with interquartile range. Statistical significance was determined using the Mann-Whitney Test. *** represent P values less than 0.001.

**Supplementary Figure 9. In patients treated with Bortezomib-based therapy, RNA expression of ST3GAL4/ST3GAL6 and FUT7 >median correlates with inferior PFS and RD while in patients treated with non-Bortezomib-based therapy, RNA expression of ST3GAL4/ST3GAL6 and FUT7 > median correlates with inferior PFS.** (A) Kaplan-Meier estimates of PFS (A) and RD (B) in Bortezomib-treated MM patients according to glycogene mRNA expression. Kaplan-Meier estimates of PFS (C) and RD (D) in non Bortezomib-treated MM patients according to glycogene mRNA expression. Blue line ST3GAL4/ST3GAL6 and FUT7 > median, red line remaining patients.

**Supplementary Figure 10. External validation of PFS estimates from the CoMMpass trial** **in the GSE2658 dataset**. Kaplan-Meier estimates of PFS in MM patients with RNA expression of either *ST3Gal-6 or ST3Gal-4* and *FUT7* greater than the median (red) and the remainder of the patients (green) show inferior overall progression free survival times (p-value 0.05), with a Hazard ratio of 1.49. X-axis represents time to progression in weeks and Y-axis represents proportion of patients without progression.
