# Supplementary figures and images for: E-selectin ligands recognised by HECA452 induce drug resistance in myeloma, which is overcome by the E-selectin antagonist, GMI-1271
Source: Leukemia. 2017 May 30;31(12):2642–51. doi: 10.1038/leu.2017.123 (PMC5729350; doi:10.1038/leu.2017.123)

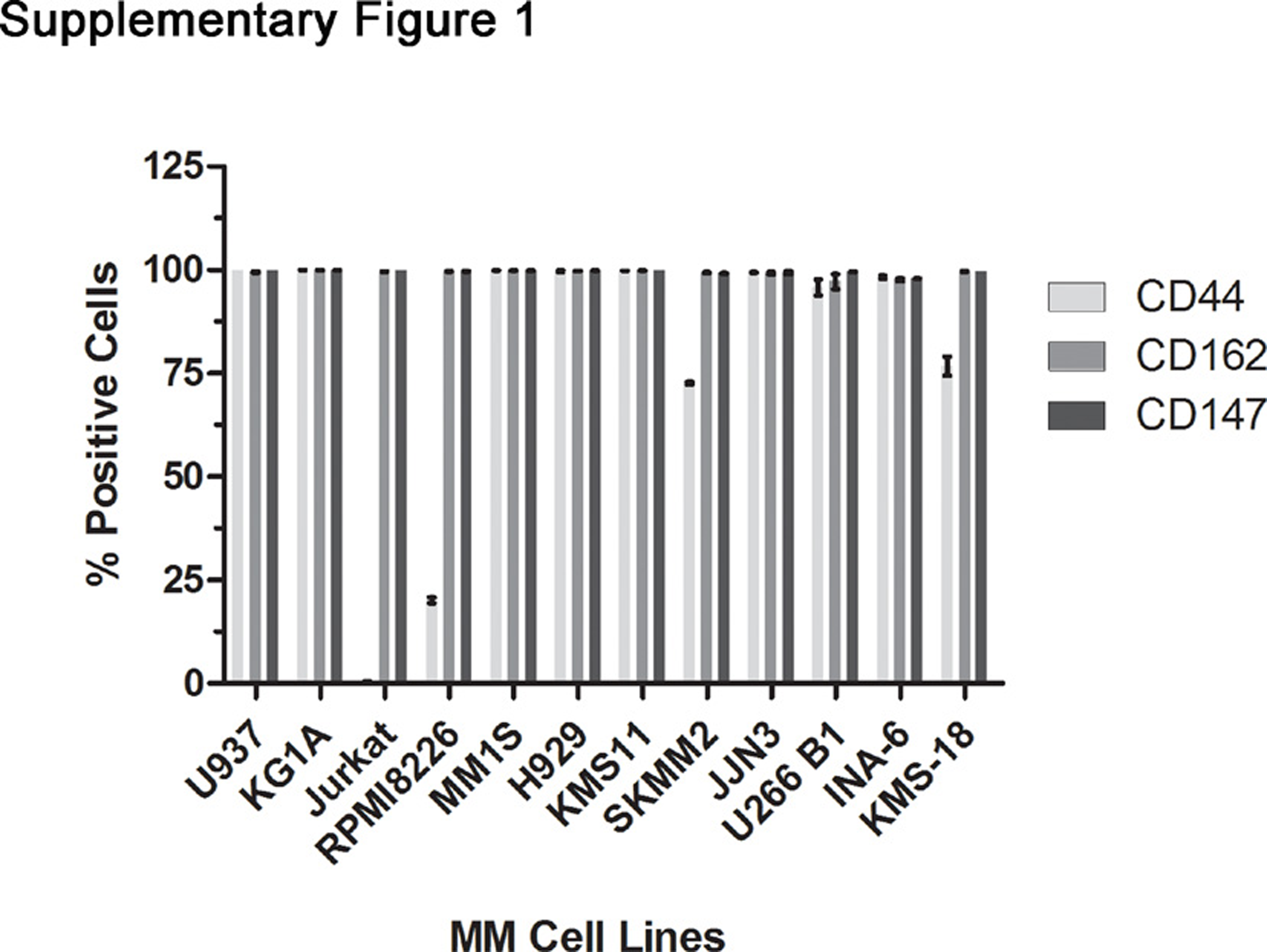

Supplement: Supplementary Figure S1 [file leu2017123x2.tif]

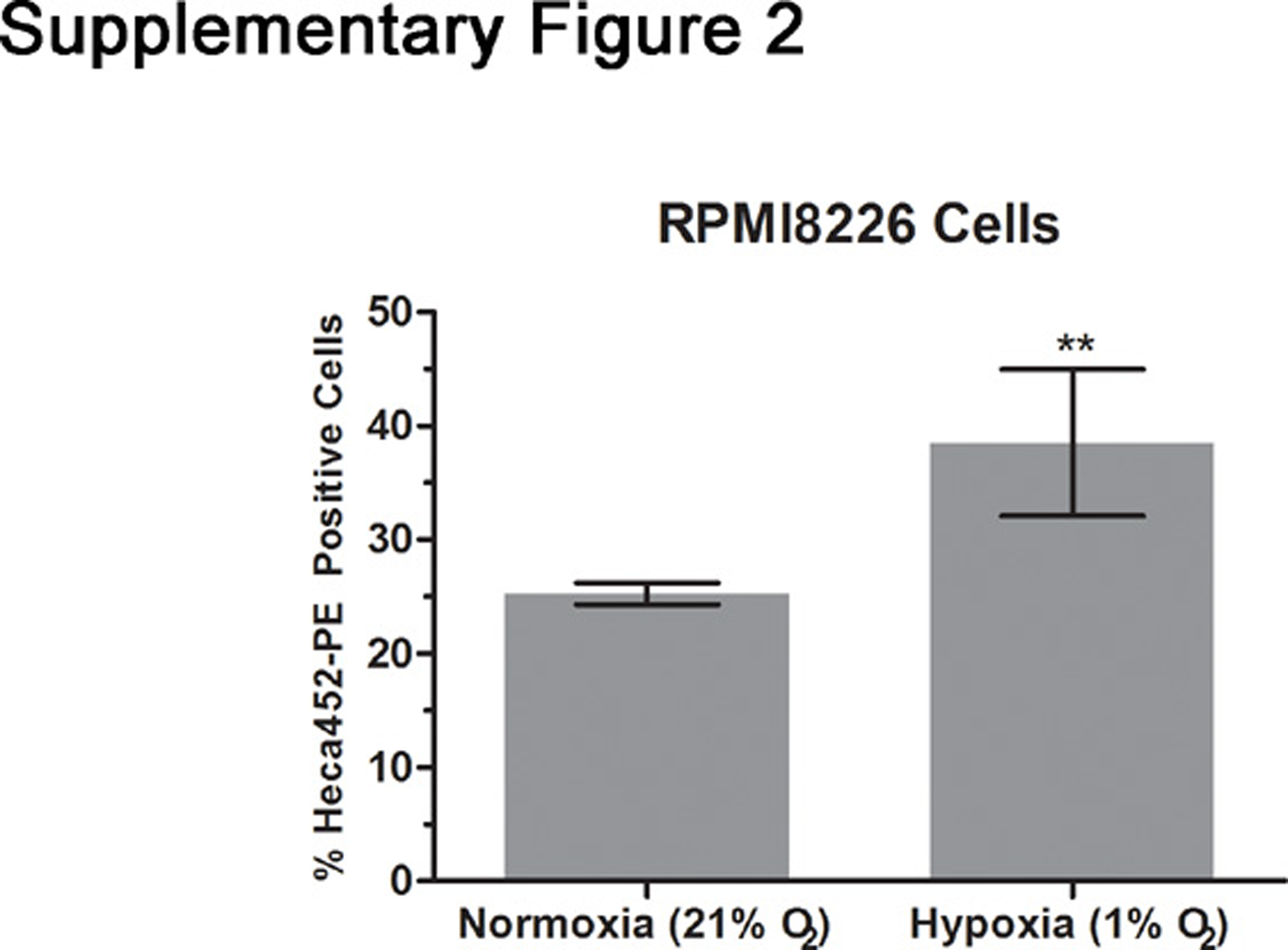

Supplement: Supplementary Figure S2 [file leu2017123x3.tif]

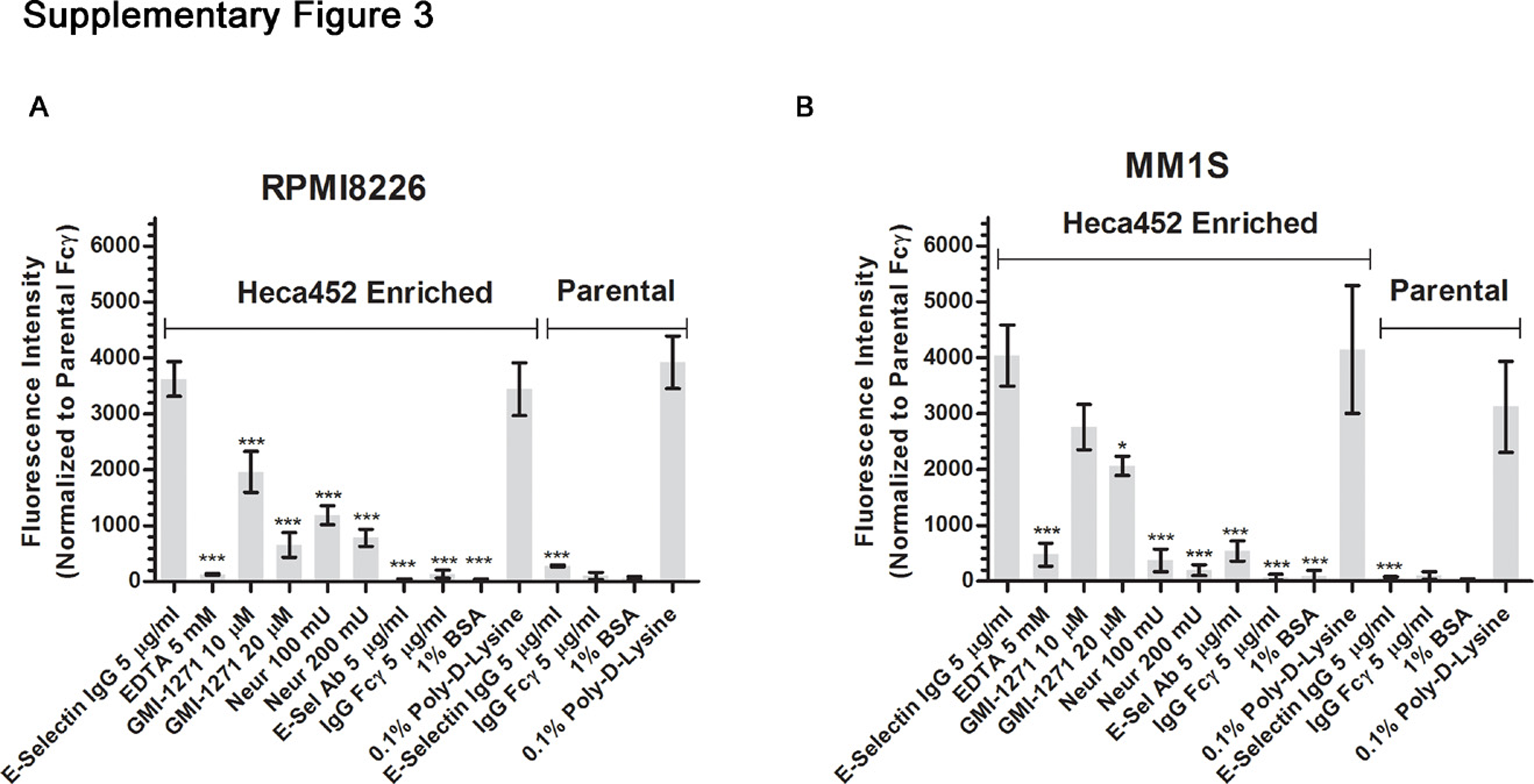

Supplement: Supplementary Figure S3 [file leu2017123x4.tif]

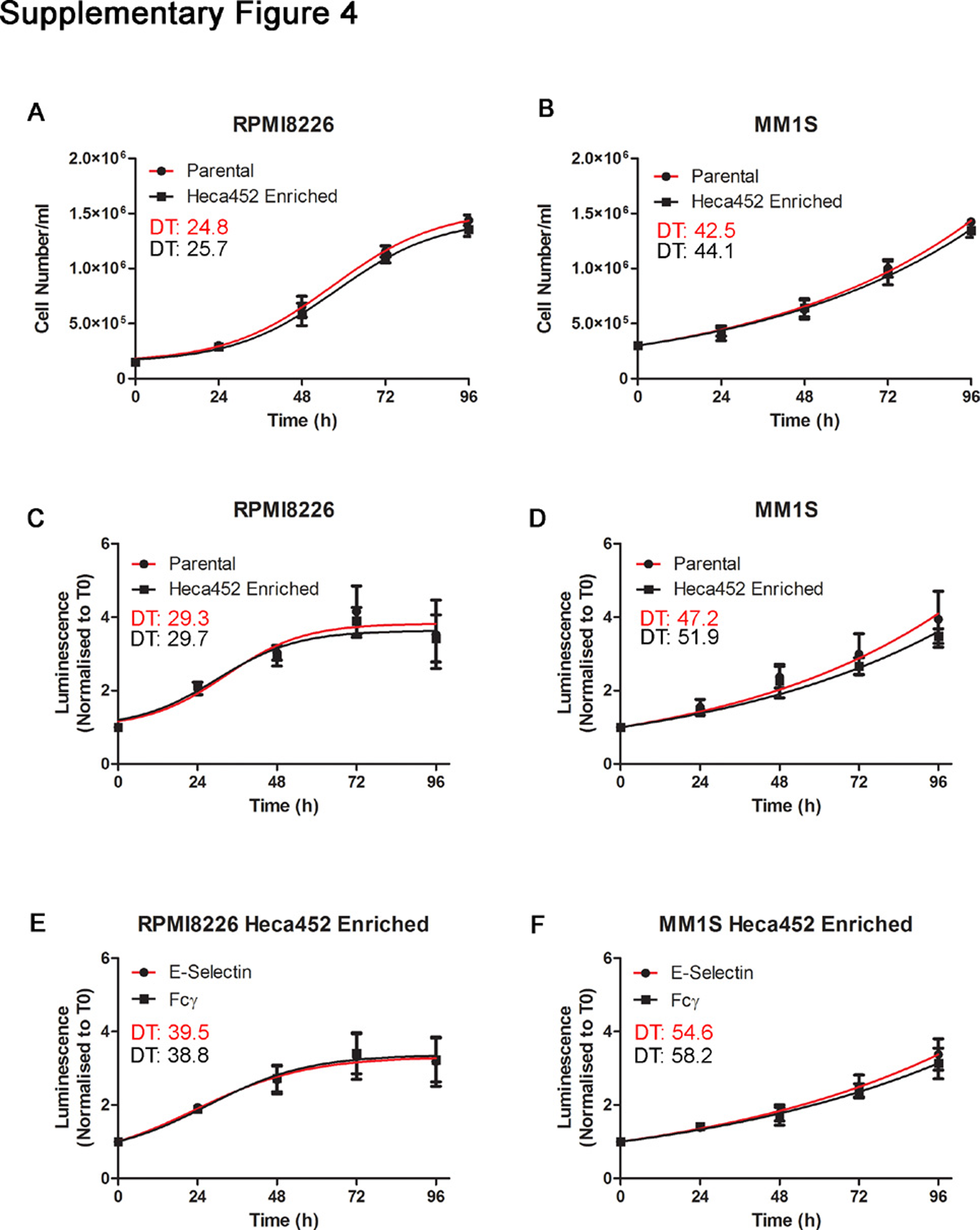

Supplement: Supplementary Figure S4 [file leu2017123x5.tif]

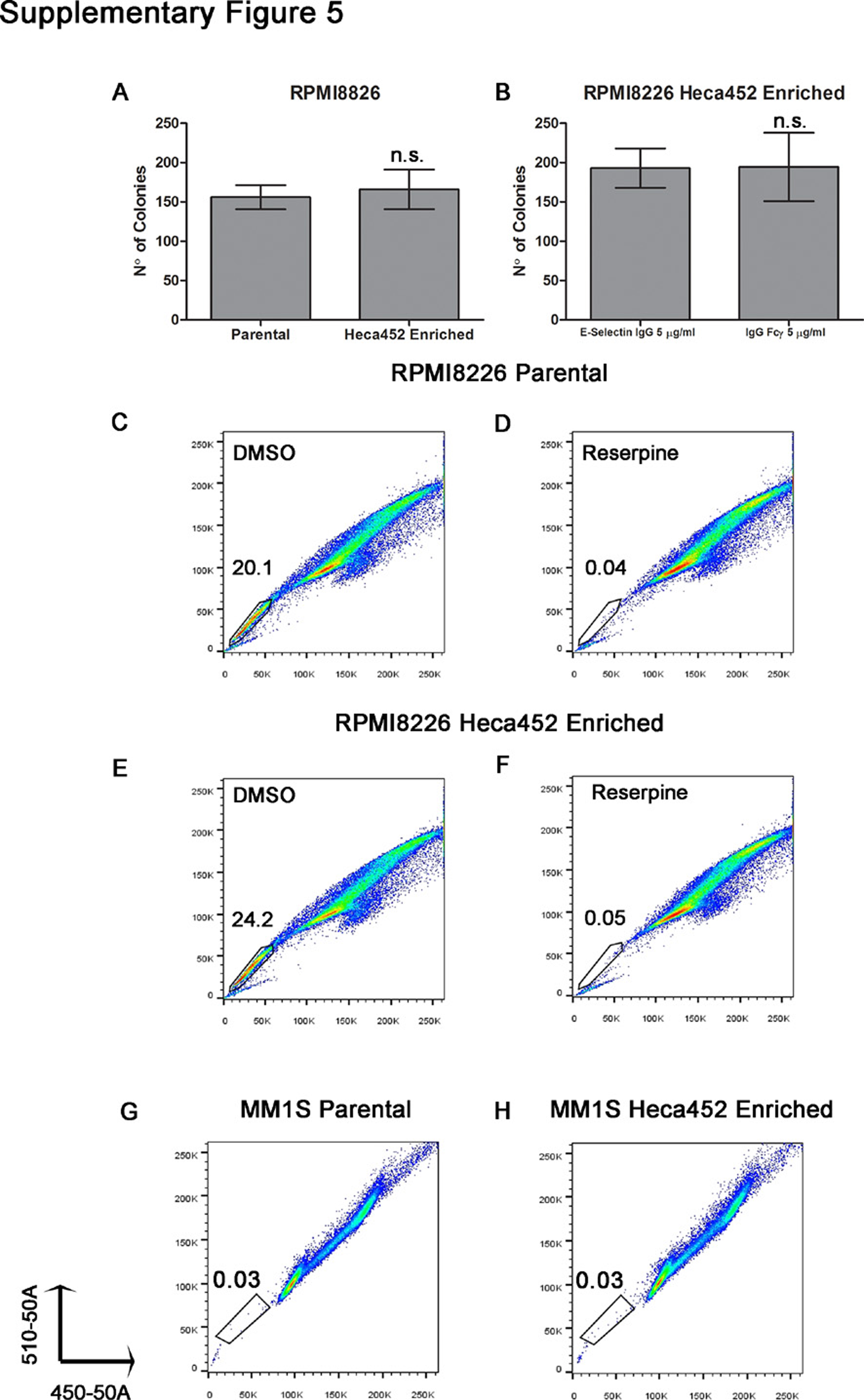

Supplement: Supplementary Figure S5 [file leu2017123x6.tif]

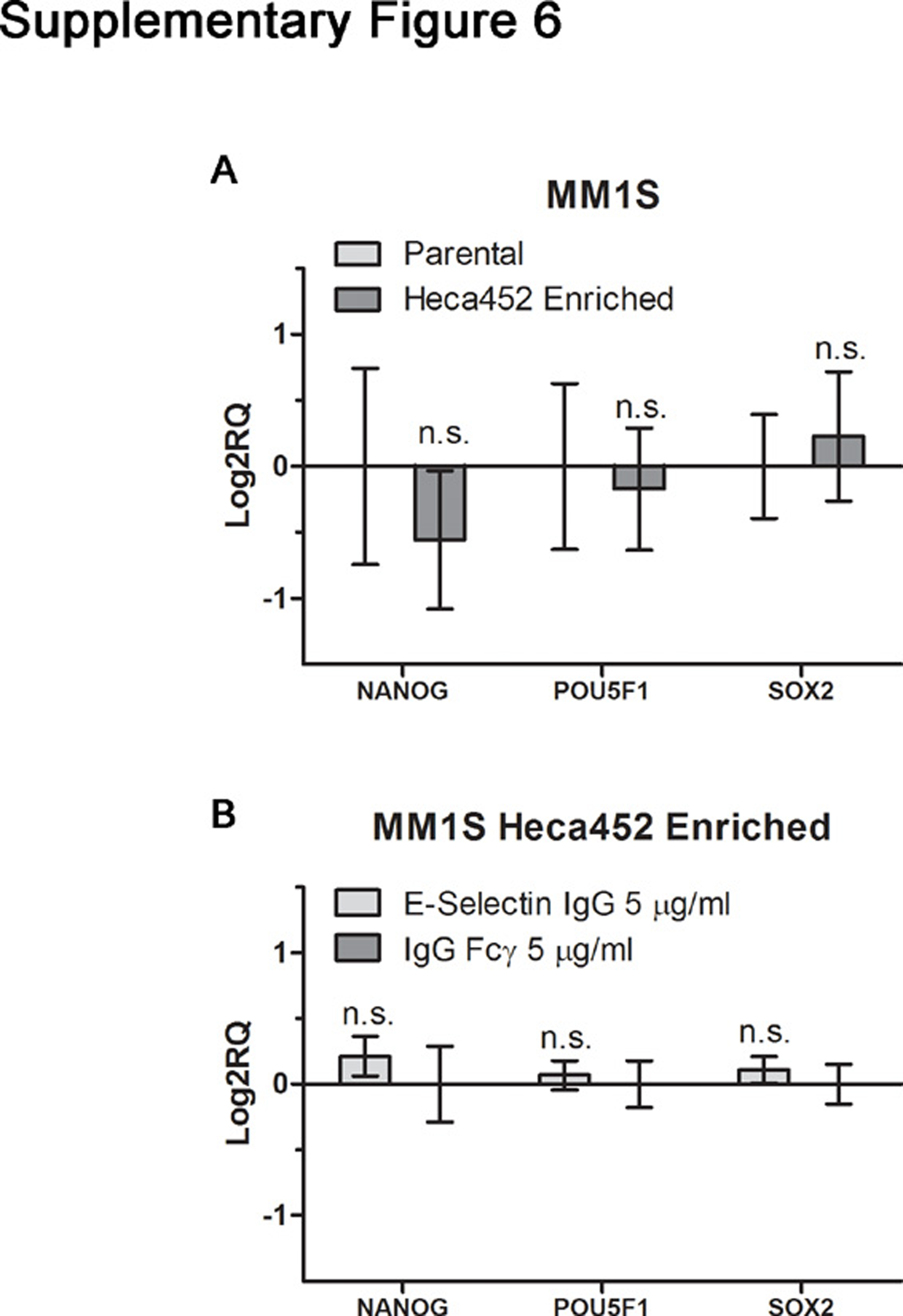

Supplement: Supplementary Figure S6 [file leu2017123x7.tif]

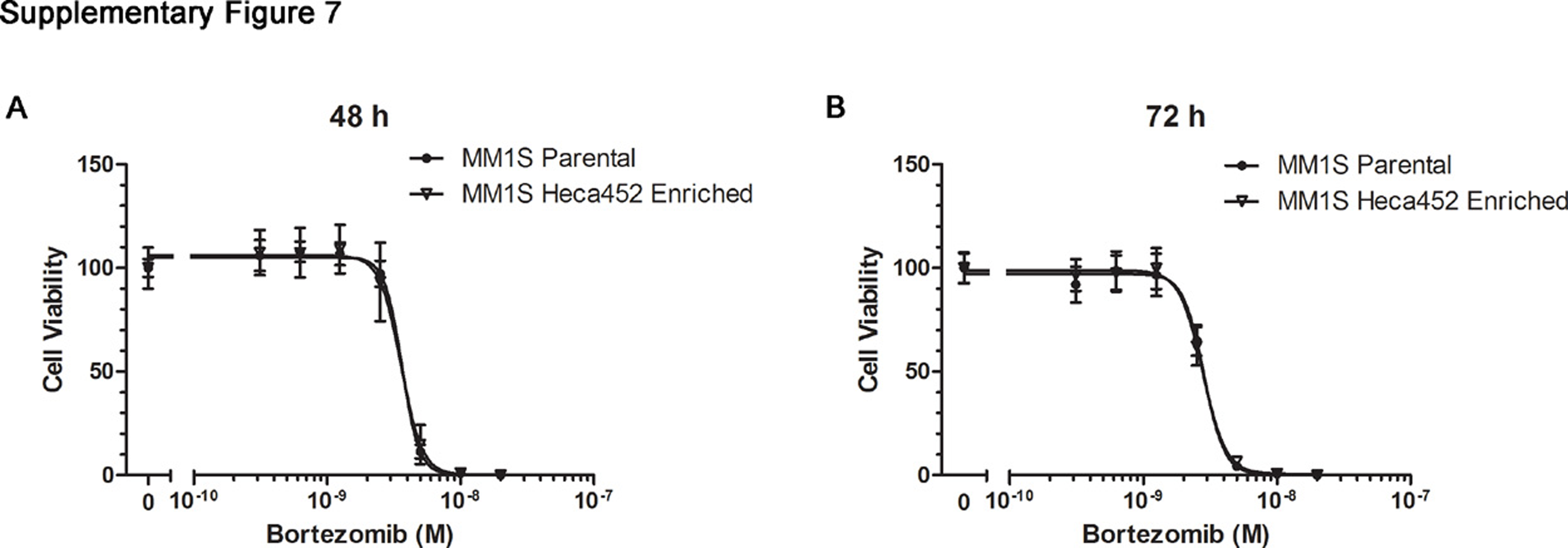

Supplement: Supplementary Figure S7 [file leu2017123x8.tif]

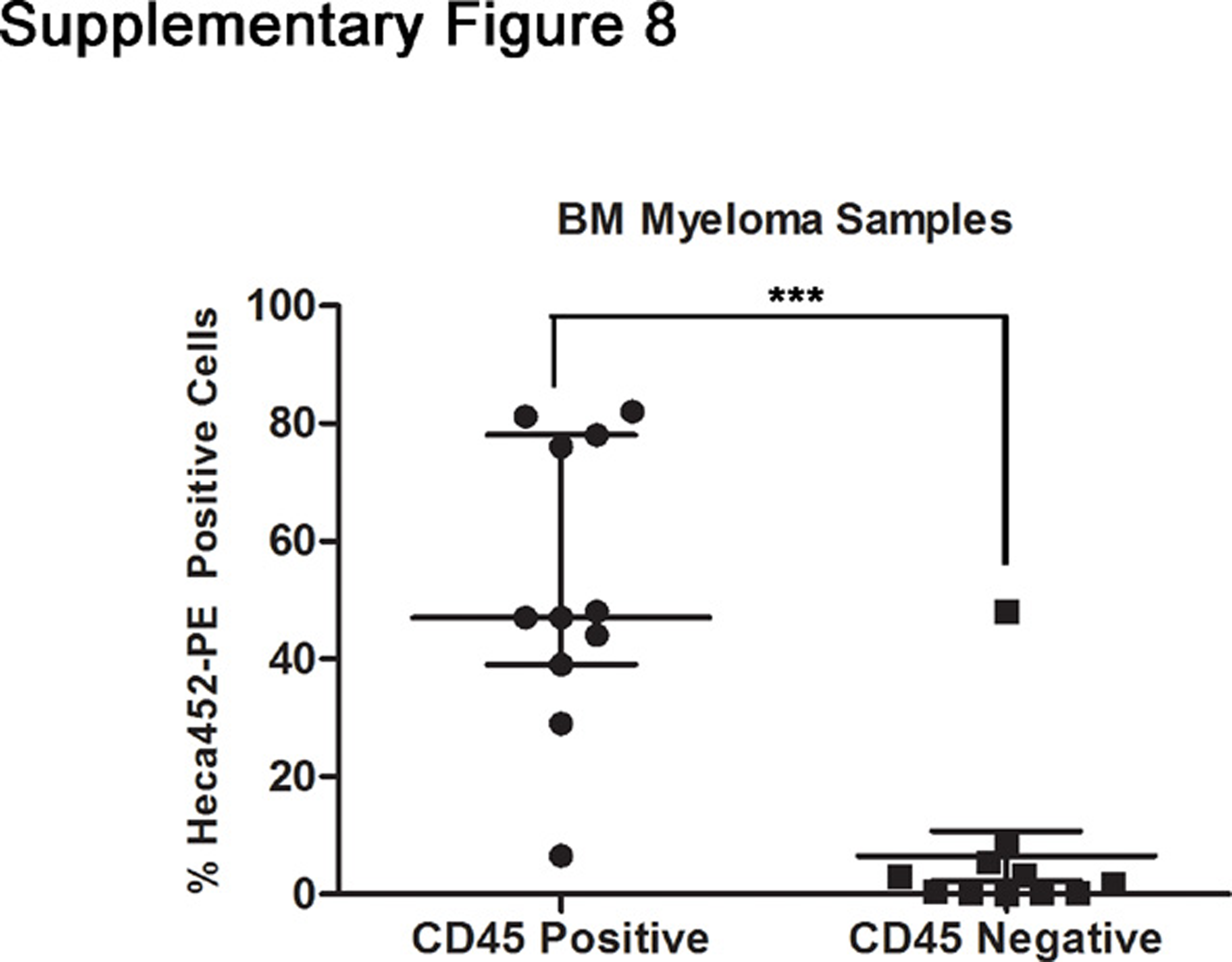

Supplement: Supplementary Figure S8 [file leu2017123x9.tif]

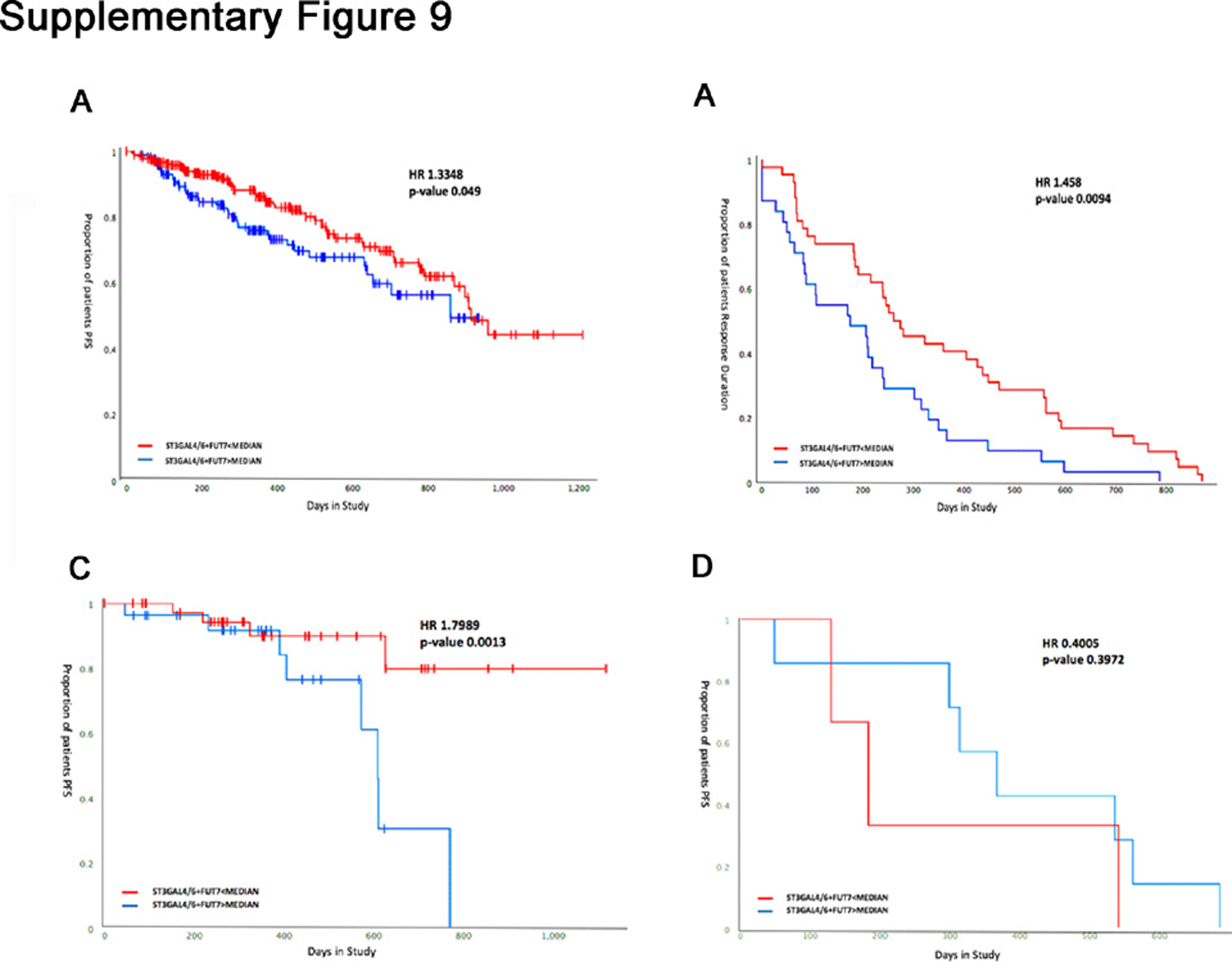

Supplement: Supplementary Figure S9 [file leu2017123x10.tif]

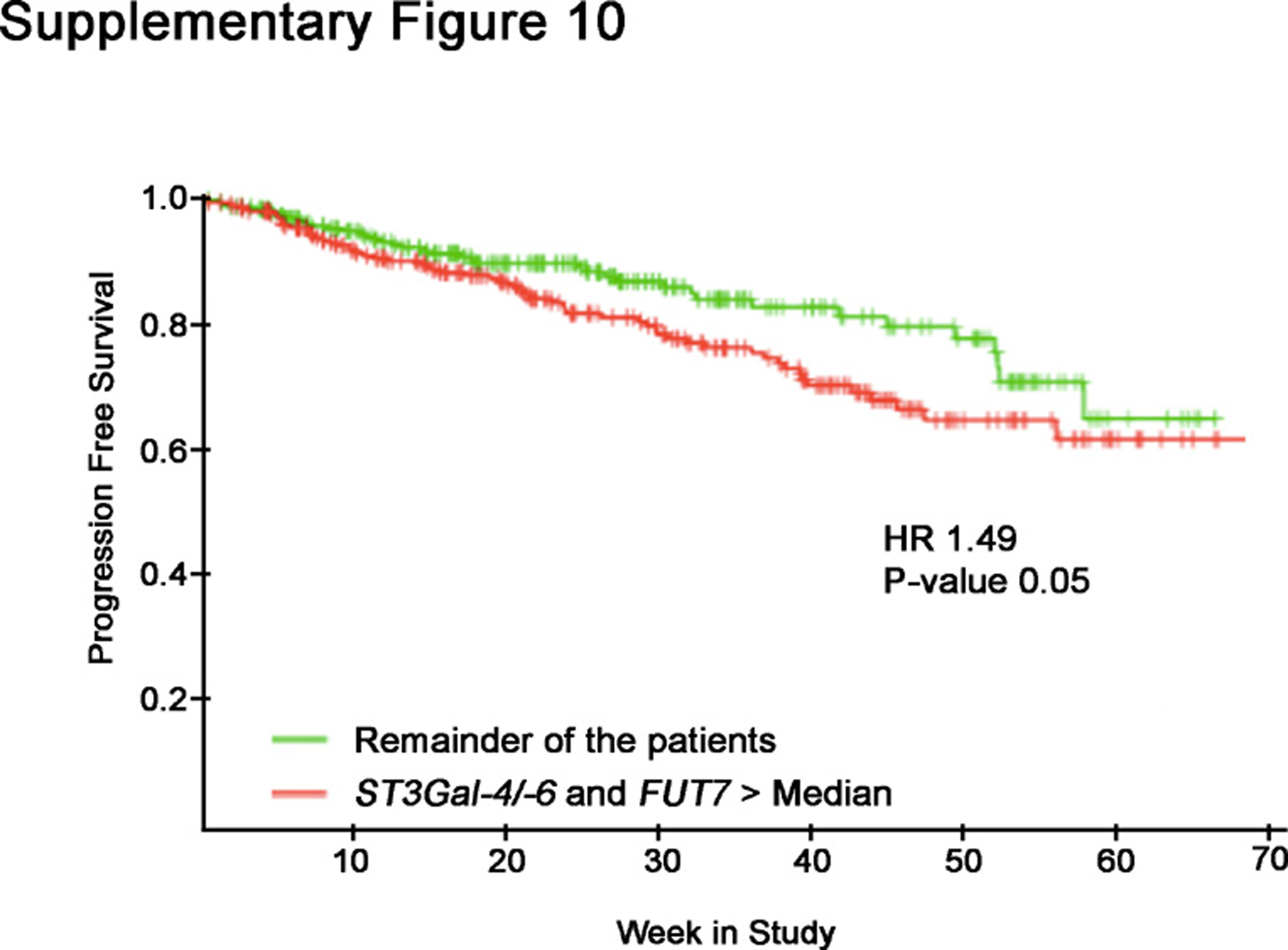

Supplement: Supplementary Figure S10 [file leu2017123x11.tif]
